# Orbital metastasis of squamous cell cervical cancer: A case report and review of literature

**DOI:** 10.1016/j.gore.2020.100689

**Published:** 2020-12-19

**Authors:** Ana Mendia, Chintan P. Shah, Joseph R. Grajo, Anthony Yachnis, Anamaria Yeung, Joel Cardenas-Goicoechea, Martina Murphy

**Affiliations:** a Department of Obstetrics and Gynecology, University of Florida, PO BOX 100294, Gainesville, FL 32610, USA; b Division of Hematology and Oncology, 1600 SW Archer Road, PO BOX 100278, Gainesville, FL 32610, USA; c Department of Radiology, University of Florida College of Medicine, PO Box 100374, Gainesville, FL 32610, USA; d Department of Pathology, University of Florida College of Medicine, PO Box 100275, Health Science Center, Gainesville, Fl 32610, USA; e Department of Radiation Oncology, University of Florida College of Medicine, Gainesville, FL 32610, USA; f Division of Gynecology Oncology, Department of Obstetrics and Gynecology, University of Florida College of Medicine University of Florida, PO BOX 100294, Gainesville, FL 32610, USA

**Keywords:** Cervical cancer, Orbital Metastasis, Visual changes, Blindness

## Abstract

Common sites of metastatic disease seen in cervical cancer most often include the lungs and liver. Orbital metastasis secondary to cervical carcinoma is a rare form of metastatic disease. We report a 73-year-old woman who presented with ocular symptoms found to be secondary to orbital metastasis of cervical cancer. She underwent palliative radiation to the orbit and pelvis followed by systemic chemotherapy with carboplatin, paclitaxel, and bevacizumab. Prompt intervention was able to salvage her vision and improve her quality of life significantly. We identified 5 similar reported cases in which orbital metastasis was diagnosed simultaneously at the time of cervical cancer diagnosis. In these five cases, patients were treated with a combination of radiation and chemotherapy. Our case demonstrates an unusual presentation of isolated orbital metastatic disease secondary to squamous cell carcinoma of the cervix. Physicians should be aware that cervical cancer may metastasize to the eye leading to vision loss, and prompt intervention may be able to salvage one’s vision and improve quality of life.

## Highlights

• Even though very rare, cervical cancer can present as isolated metastasis to orbit.
• Should get imaging and biopsy for confirmation when suspected.
• Treatment consists of radiation therapy, steroids, chemotherapy, and/or surgery.
• Prompt intervention may salvage vision and improve quality of life.

## 1 Background

Worldwide, cervical cancer is the fourth most common malignancy diagnosed in women with approximately 500,000 new cases diagnosed annually and remains the most common gynecologic malignancy (Ferlay et al., 2018). The principal subtype of cervical cancer is squamous cell carcinoma, accounting for approximately 70% of cases (Ries et al., 2007). Cervical cancer is known to be locally invasive with the most common spread via direct extension in the pelvis. As the tumor grows, lymphatic spread to the pelvic and retroperitoneal lymph nodes is commonly observed (Bhandari et al., 2016). While rare, distant metastases resulting from hematogenous dissemination can occur, with the lungs, liver, and bone most frequently affected (Bhandari et al., 2016). Here, we present a rare case of a woman with squamous cell carcinoma of the cervix with metastasis to the orbit.

## 2 Case

A 72-year-old Caucasian female with past medical history significant for remote history of supracervical hysterectomy for uterine fibroids presented to the emergency room with complaints of diplopia, left eye pain, and gradually worsening left-sided headache of one week duration. She had also been experiencing mild intermittent vaginal bleeding for several months. Ophthalmologic exam revealed left lateral rectus muscle restriction and was otherwise unremarkable. She didn’t have history of abnormal pap smear; however, her last pap smear had been over thirty years ago. Pelvic examination revealed a large necrotic mass occupying the vaginal vault approximately 7 cm in size with parametrial involvement appreciated on bimanual exam. Remainder of physical examination was unremarkable. Past medical history was only significant for a 45-pack year history of smoking and history of iron deficiency anemia related to GI arteriovenous malformation.

**Fig. 1:**
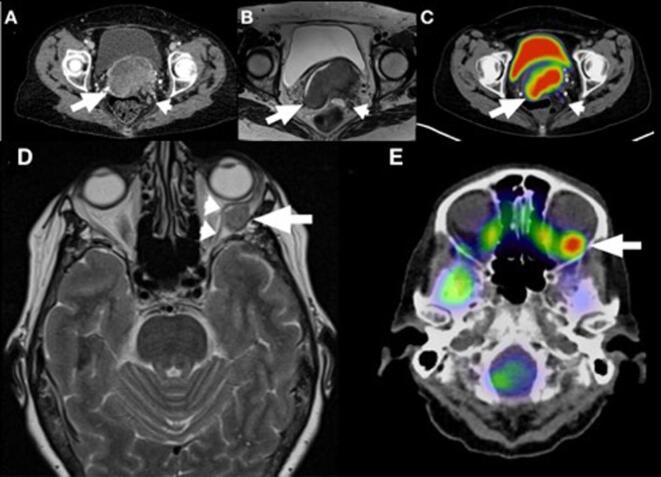
(A–C) Contrast-enhanced axial CT of the pelvis (A) shows a large necrotic tumor within the cervix (arrow) with mesorectal lymphadenopathy (arrowhead). Subsequent axial T2-weighted MRI for local staging of the cervical tumor (arrow) shows left parametrial extension with invasion into the mesorectal fat and associated adenopathy (arrowhead). PET/CT (C) performed at the same time as in figure shows hypermetabolic activity within the tumor (arrow) and lower level uptake in the enlarged mesorectal lymph node (arrowhead). (D,E): Axial T2-weighted MRI of the brain (D) demonstrates a 1.4 cm mass within the left lateral orbit (arrow), which results in superomedial displacement of the left lateral rectus muscle (arrowheads), accounting for the patient's clinical presentation of lateral rectus palsy. A subsequent PET/CT (E) shows avid FDG uptake within the presumed metastatic lesion.

Biopsy of the cervical mass revealed an infiltrating poorly differentiated squamous cell carcinoma. MRI of the pelvis revealed a large mass in the expected area of the cervix measuring 6.7 × 3.6 × 7.3 cm in size with extension through the left lateral parametrium into the mesorectal fat, as well as a 2 cm long segment of abutment to the posterior bladder wall, partial encasement of the urethra, and invasion into the anterior rectal wall (Fig. 1A–C). MRI of the orbit was performed, which revealed an enhancing mass measuring 1.2 × 1.1 × 1.4 cm in the lateral aspect of the left orbit displacing the left lateral rectus muscle superiorly and medially resulting in lateral rectus palsy (Fig. 1D and E). A transvaginal ultrasound revealed a 6.9 cm solid mass replacing the expected location of the cervix and/or vaginal cuff. A contrast enhanced computerized tomography (CT) scan revealed a 5.2 × 8.1 cm mass in the region of the cervix extending into the vagina. Laboratory work-up was significant for anemia, with hemoglobin of 10.1 and hematocrit of 30.6. A basic metabolic panel was unremarkable with a normal creatinine of 0.67.

To further evaluate for metastasis, a whole-body positron emission tomography (PET) scan revealed intense uptake in the cervix with a maximum standard uptake value (SUV) of 20.3 as well as uptake of two adjacent pelvic lymph nodes in the left pelvis lateral to the cervix with SUV of 5.0. There was also asymmetrical FDG uptake in the left posterior orbit and no evidence of uptake in other distant organs such as liver, lung, or distant lymph nodes.

**Fig. 2:**
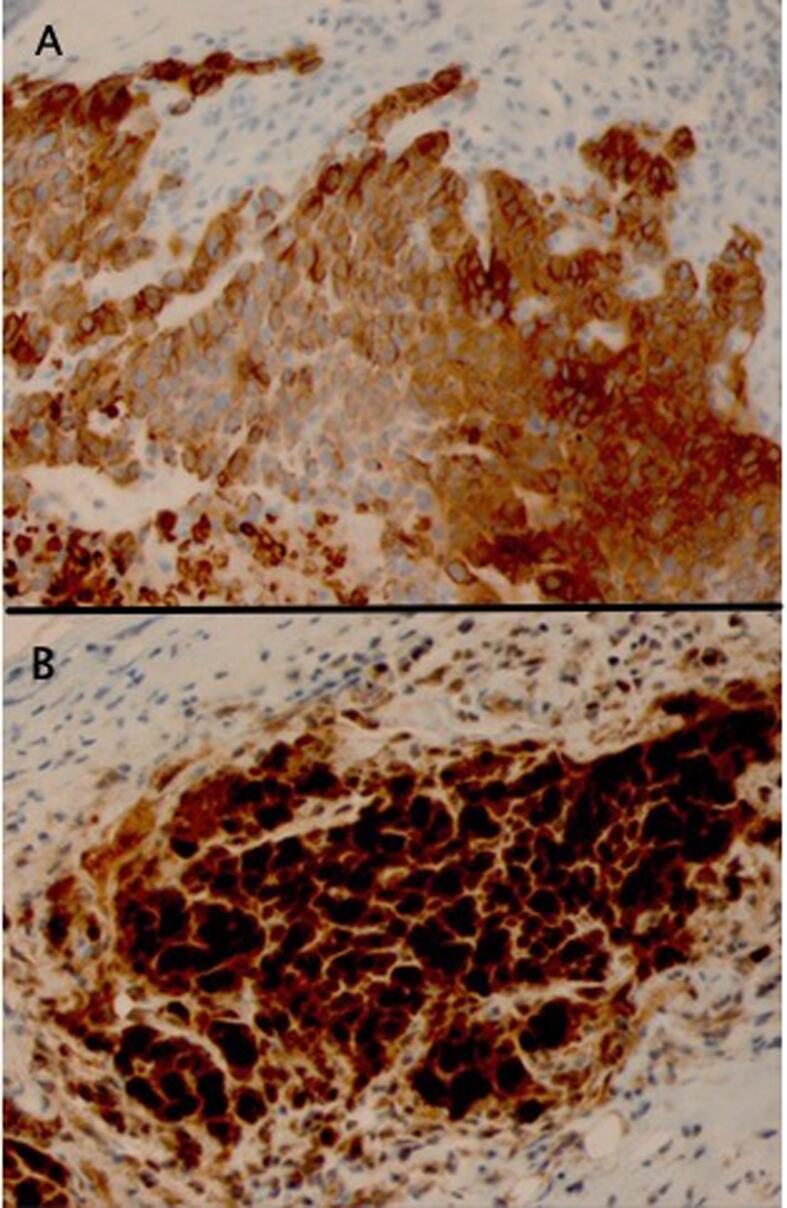
(A) Immunohistochemical study for cytokeratin 5/6 showing diffuse, strong cytoplasmic reactivity of tumor cells. (B) Immunohistochemical study for p16 showing diffuse, strong nuclear reactivity of tumor cells. These results also support a poorly differentiated squamous cell carcinoma.

Orbital biopsy was performed and revealed poorly differentiated carcinoma, consistent with nonkeratinizing squamous cell carcinoma. Immunohistochemical stains performed were positive for CK5/6 and P16 (Fig. 2A and B) and negative for P40 supporting HPV associated malignancy in this patient with a cervical primary neoplasm. Given that the biopsy confirmed metastasis to the left orbit, she was ultimately diagnosed with Stage IVB squamous cell carcinoma of the cervix.

She completed palliative 3D conformal radiation to the left retroorbital mass and pelvis with 30 Gy over 10 fractions for both locations. Concurrent radiosensitizer chemotherapy wasn’t administered. She tolerated radiation well with mild vulvar and perineal skin toxicity, which subsequently healed without complications. Following completion of radiation, chemotherapy was initiated with carboplatin AUC 6, paclitaxel 175 mg/m2, and bevacizumab 15 mg/kg every 3 weeks. Carboplatin was selected opposed to cisplatin (unlike GOG 240) due to favorable toxicity profile.

**Fig. 3:**
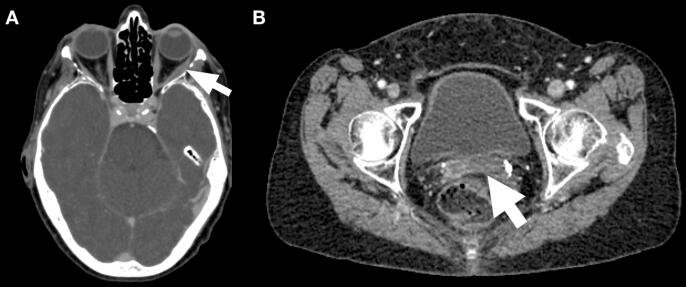
(A,B) Axial post-contrast CT images demonstrate treatment response. (A) Residual swelling of the left lateral rectus muscle (arrow) with resolution of previously visualized discrete mass. (B) Likewise, marked decrease in size of the primary cervical tumor (arrow).

She completed a total of six planned cycles of chemotherapy. Subsequently surveillance was planned and repeat imaging with contrast enhanced CT scan after completing six cycles of chemotherapy showed a positive response (Fig. 3A and B). She tolerated chemotherapy without significant side effects, and her double vision had resolved since radiation treatment to the left orbit. About 4 months after completing chemotherapy, she was found to have diffuse brain metastases detected on MRI. She received whole brain radiation therapy with 30 Gy over 10 fractions. Shortly after that she developed right cerebellar intracranial hemorrhage from hemorrhagic metastasis, obstructive hydrocephalus and bilateral cerebellar tonsillar herniation leading to brain stem compression and death.

## 3 Discussion

Orbital metastases account for only 3–5% of all orbital tumors (Amemiya et al., 2002). Malignancies most commonly associated with orbital metastases include lung, breast, and prostate cancers (Amemiya et al., 2002, Shields et al., 2004). Cervical cancer is an extremely rare cause of orbital metastasis (Amemiya et al., 2002). Here, we report an unusual case of newly diagnosis primary cervical cancer with the orbit as the only site of distant metastasis.

In the United States, cervical cancer is the third most common cancer diagnosis and cause of death among gynecologic cancers, and approximately 14,000 new cases of invasive cervical cancer occur each year (Siegel et al., 2020). The incidence is expected to decrease in upcoming years with the use of HPV vaccination. In the US, approximately 44% of patients present at an early stage due to the widespread utilization of cervical cancer screening (https://www.cancer.org/content/dam/cancer-org/research/cancer-facts-and-statistics/annual-cancer-facts-and-figures/2020/cancer-facts-and-figures-2020.pdf). Current guidelines recommend routine screening for cervical cancer with pap smear and high risk HPV testing until the age of 65 (Solomon et al., 2007). In this case, our patient had a supracervical hysterectomy thirty years prior to presentation. Following surgery, the patient was lost to gynecological follow-up and never completed routine cervical cancer screening. Cervical cancer usually spreads by local extension to contiguous areas and by the lymphatic drainage to the lymph nodes (Bhandari et al., 2016). Early stage cervical cancer is frequently asymptomatic and may remain undiagnosed until it spreads locally or leads to distant metastasis. Approximately 15 percent of patients have distant metastases at the time of diagnosis. Common sites for distant metastasis via hematogenous spread include lung and liver. Orbital metastasis is rarely seen in cervical malignancies (Ferlay et al., 2018). Spread to the orbit appears to occur via the venous plexus of Batson and the inner vertebral plexus (Tangjitgamol et al., 2004).

We performed a detailed PUBMED search for cases involving cervical cancer with orbital metastasis. We identified 5 cases similar to our case in which orbital metastasis was simultaneously diagnosed at the time of cervical cancer diagnosis (Table 1) (Nash et al., 2017, Nair et al., 2015, Shibeeb et al., 2014, Singh et al., 2011, Hertzanu et al., 1987). In these five cases, the most common site of metastasis within the orbit was the choroid (Nair et al., 2015, Shibeeb et al., 2014). This is likely because the choroid is a highly vascular structure. Pathology from these similar cases included two cases with adenocarcinoma of the cervix (Nash et al., 2017, Nair et al., 2015) and three cases with squamous cell carcinoma of the cervix (Shibeeb et al., 2014, Hertzanu et al., 1987). In three of the five similar cases, there was no evidence of other distant metastasis present (Nash et al., 2017, Singh et al., 2011, Hertzanu et al., 1987). Furthermore, among these 5 cases, the disease followed a progressive course with three patients reportedly dead of disease within 1–3 months following diagnosis (Nash et al., 2017, Nair et al., 2015, Shibeeb et al., 2014) and the remaining two patients with unknown outcomes (Singh et al., 2011, Hertzanu et al., 1987).

**Table 1:** Details of cases reported in literature.

Author, Year of Publication	Age	Histology	Presenting Symptoms	Site within orbit	Timing of metastasis from original diagnosis	Treatment of Orbital Metastasis	Other sites of metastasis	Survival
Hertzanu et al. (1987)	44	Moderately differentiated, non-keratinizing squamous cell carcinoma	Proptosis, visual Loss, diplopia	Orbit (right, intraconal + extraconal)	Simultaneously	Chemotherapy	None	Unknown
Singh et al. (2011)	50	Keratinizing squamous cell carcinoma	Ocular edema	Orbit (left, medial canthus)	Simultaneously	Chemotherapy, Radiation	None	Unknown
Shibeeb et al. (2014)	52	Squamous cell Carcinoma, Large Cell Type	B/L visual loss	B/L Choroids	Simultaneously	Radiation	Liver, Bone	DOD − 3 months following eye symptoms
Nair et al. (2015)	45	Poorly differentiated adenocarcinoma	Visual Loss, retinal detachment	Choroid (right)	Simultaneously	None – Declined	Lung, Supraclavicular lymph node, skull, femur	DOD − 2 months following eye symptoms
Nash et al. (2016)	Unknown	High grade adenocarcinoma	Proptosis, Eyelid & ocular edema,	Orbital subperiosteal space (left)	Simultaneously	Surgery, declined further treatment	None	DOD − 1 month following eye symptoms
Current Case	73	Poorly differentiated squamous cell carcinoma	Eye Pain, Diplopia, EOM restriction	Orbit (left)	Simultaneously	Chemotherapy, Radiation	None	Alive 10 months following diagnosis

Abbreviations: B/L, bilateral; EOM, extraocular muscle; DOD: Dead of Disease.

The treatment of metastatic cervical cancer includes palliative chemotherapy and radiation. Given the rarity of orbital metastatic disease from cervical cancer, biopsy confirmation is important. Our patient had no evidence of distant disease in any other sites besides the orbit and thus biopsy confirmation was obtained to confirm metastatic cervical cancer. The approach to orbital metastasis from cervical cancer is not standardized given its rare nature. Treatment is palliative for symptom control and may consist of radiation therapy, steroids, chemotherapy, and/or surgery. Surgical excision may increase morbidity and lead to poor quality of life but can be considered if radiation therapy fails to control symptoms. Management of the five cases similar to our own varied significantly. Two patients declined further treatment and died of their disease within one and two months of diagnosis (Nash et al., 2017, Nair et al., 2015). A third patient received palliative external beam radiotherapy to the orbits bilaterally and subsequently died three months following initial presentation (Shibeeb et al., 2014). The final two patients did not have disease outcomes reported in the literature. Of these, one patient was started on palliative chemotherapy with carboplatin and paclitaxel with plans for palliative radiation therapy (Singh et al., 2011) and the second patient received palliative chemotherapy only (Hertzanu et al., 1987). Our patient was treated with palliative radiation to her ocular metastatic disease resulting in marked improvement in her ocular symptoms. Her treatment course also included radiation to the pelvis followed by chemotherapy. At the time of publication of this manuscript, our patient has survived 10 months since initial diagnosis.

In summary, our case demonstrates an unusual case of an elderly patient presenting with sudden visual changes and found to have orbital metastasis from cervical cancer. Physicians should be aware that cervical cancer may metastasize to the eye and can lead to significant morbidity and substantial effects on quality of life. When ocular symptoms such as eye protrusion, eye pain, and visual changes appear suddenly, malignancy should be suspected. When metastasis from cervical cancer is suspected in a patient with an isolated orbital mass without any evidence of other distant metastasis, biopsy should be performed for confirmation. The presence of orbital metastasis may suggest a poor prognosis as the majority of patients reported in the literature died within a year of diagnosis, however prompt intervention may be able to salvage one’s vision and improve quality of life. A reasonable approach for localized metastasis is targeted radiation followed by systemic chemotherapy or clinical trial.

## CRediT authorship contribution statement

**Ana Mendia:** Conceptualization, Writing - original draft, Writing - review & editing. **Chintan P. Shah:** Conceptualization, Writing - original draft, Writing - review & editing. **Joseph R. Grajo:** Resources, Writing - review & editing. **Anthony Yachnis:** Resources, Writing - review & editing. **Anamaria Yeung:** Resources, Writing - review & editing. **Joel Cardenas-Goicoechea:** Resources, Writing - review & editing. **Martina Murphy:** Conceptualization, Writing - original draft, Writing - review & editing.

## Declaration of Competing Interest

The authors declare that they have no known competing financial interests or personal relationships that could have appeared to influence the work reported in this paper.
